# Effect of Organo-Silanes Structure on the Properties of Silane-Crosslinked Membranes Based on Cardo Polybenzimidazole PBI-O-PhT

**DOI:** 10.3390/membranes12111078

**Published:** 2022-10-31

**Authors:** Anna A. Lysova, Igor I. Ponomarev, Kirill M. Skupov, Elizaveta S. Vtyurina, Kirill A. Lysov, Andrey B. Yaroslavtsev

**Affiliations:** 1 Kurnakov Institute of General and Inorganic Chemistry RAS, Leninskii Prospect, 31, 119071 Moscow, Russia; 2 A.N. Nesmeyanov Institute of Organoelement Compounds of Russian Academy of Sciences, Vavilova St., 28, bld. 1, 119334 Moscow, Russia

**Keywords:** membrane, polyelectrolyte, polybenzimidazole, covalent crosslinking, silanol crosslinking, proton conductivity, gas permeability, mechanical properties, HT-PEMFC, phosphoric acid

## Abstract

Polybenzimidazoles (PBI) doped with phosphoric acid (PA) are promising electrolytes for medium temperature fuel cells. Their significant disadvantage is a partial or complete loss of mechanical properties and an increase in hydrogen permeability at elevated temperatures. Covalent silanol crosslinking is one possible way to stabilize PBI membranes in the presence of PA. Three organo-substituted silanes, namely (3-Bromopropyl)trimethoxysilane (SiBr), trimethoxy [2-(7-oxabicyclo [4.1.0]hept-3-yl)ethyl]silane (Si-biC) and (3-Glycidyloxypropyl)trimethoxysilane (KH 560), were used as covalent crosslinkers of PBI-O-PhT in order to determine the effect of the silane structure and crosslinking degree on membrane properties. The crosslinking degree was 1–50%. All crosslinked membranes were characterized by impedance and IR-spectroscopy. The mechanical properties, morphology, stability and hydrogen permeability of the membranes were determined. In the case of silanes with linear substituents (SiBr, KH 560), a denser structure is formed, which is characterized by greater oxidative stability and lower hydrogen permeability in comparison to the silane with a bulk group. All the crosslinked membranes have a higher mechanical strength compared with the initial PBI-O-PhT membrane both before and after doping with PA. Despite the hardening of the polymer matrix of the membranes, their proton conductivity changes insignificantly. It was shown that cross-linked membranes can be used in fuel cells.

## 1. Introduction

Despite considerable attention being paid to environmentally friendly energy sources, to fuel cells (FCs) in particular, both the optimization of their properties and development of new materials for them remain an urgent problem. Most fuel cells are low-temperature proton exchange membrane fuel cells (LT-PEMFC), whose operating temperature usually does not exceed 100 °C. A quick start up and simplicity of operation are their main advantages. Often, sulfonated polymers such as Nafion^®^ are used as polyelectrolytes, with proton conductivity passing through a system of pores and channels [1,2]. For the high proton conductivity of such membranes, a high degree of hydration is necessary [3]. Therefore, at temperatures above 100 °C, most sulfonated polyelectrolytes lose their conductive properties due to water evaporation [4]. The operating temperature of such FCs is usually limited to 90 °C, which creates additional requirements for cooling and humidification in the system and increases the load of the catalyst to maintain the required power. It is also necessary to use high purity hydrogen, which generally leads to higher battery costs.

High temperature polymer electrolyte membrane fuel cells (HT_PEMFC), capable of operating at 120–200 °C and low humidity, are among the many promising devices for producing environmentally clean energy [5,6]. Under these conditions, the catalytic activity of platinum, its tolerance to an increase in CO impurities and fuel cell management are simplified [7,8]. One of the main types of membrane materials for their construction are polybenzimidazoles (PBI) doped with phosphoric acid (PA) [9,10,11,12,13]. These are of interest due to their high thermal stability, excellent mechanical properties and high proton conductivity at FC operating temperatures.

The doping of PBI is usually performed by soaking in phosphoric acid. The PA doping level, temperature and relative humidity are important parameters affecting the proton conductivity of PBI membranes. At the same time, a high PA doping level leads to a deterioration in mechanical strength, dimensional stability and membrane selectivity (an increase in gas permeability). In addition, the conductivity decreases due to acid leaching during FC operation, which leads to an irreversible loss of battery power and the corrosion of components. In this regard, research studies have been actively carried out to find approaches to maintain membrane stability and high levels of acid doping [14,15].

Stabilizing the conductivity of PBI membranes in the presence of phosphoric acid and reducing leaching is one of the main challenges facing the development of PBI-based materials. A possible strategy in this case is interchain covalent crosslinking. It is known that crosslinking not only improves mechanical properties but also contributes to greater oxidative stability and less deformation of the material compared with PBIs with linear structures [16,17]. Crosslinking leads to pore size limitations and a reduction in membrane gas permeability. At the same time, with an increase in the amount of crosslinking agent, the acid doping level and proton conductivity decrease [18,19]. This is also due to the fact that crosslinking involves active NH-groups which are excluded from the processes of proton transport and acid sorption [20]. Therefore, it is important to find the optimal amount of crosslinking agent that will not critically decrease conductive properties.

Potential crosslinking agents can be compounds capable of participating in the nucleophilic substitution reaction with the NH-group of polybenzimidazole, simultaneously forming bonds with different units of the PBI chain, such as di- and trihalogen-substituted aromatic compounds, ionic liquids, other polymers and many other compounds [21,22,23,24]. Organosilanes, which form siloxane –O–Si–O–Si– bond chains and can covalently bind to the NH-group of PBI, are often used [17,25]. An important advantage of using silanes for crosslinking is soft reaction conditions that do not require high temperatures.

The aim of this work was to study the effect of the silane structure and crosslinking degree on the properties of the obtained materials. Organosilanes with substituents of different structures were used as crosslinking agents for materials based on polybenzimidazole PBI-O-PhT. This polymer possesses high thermostability and oxidation stability but requires high-temperature treatment (350–400 °C) to stabilize the mechanical properties in the presence of PA, resulting in reduced acid absorption by the membrane. In this regard, as a crosslinking method, covalent silanol crosslinking under softer conditions would appear to be promising.

## 2. Materials and Methods

### 2.1. Preparation of Membranes

#### 2.1.1. PBI-O-PhT Polymer Preparation

**Figure 1:**
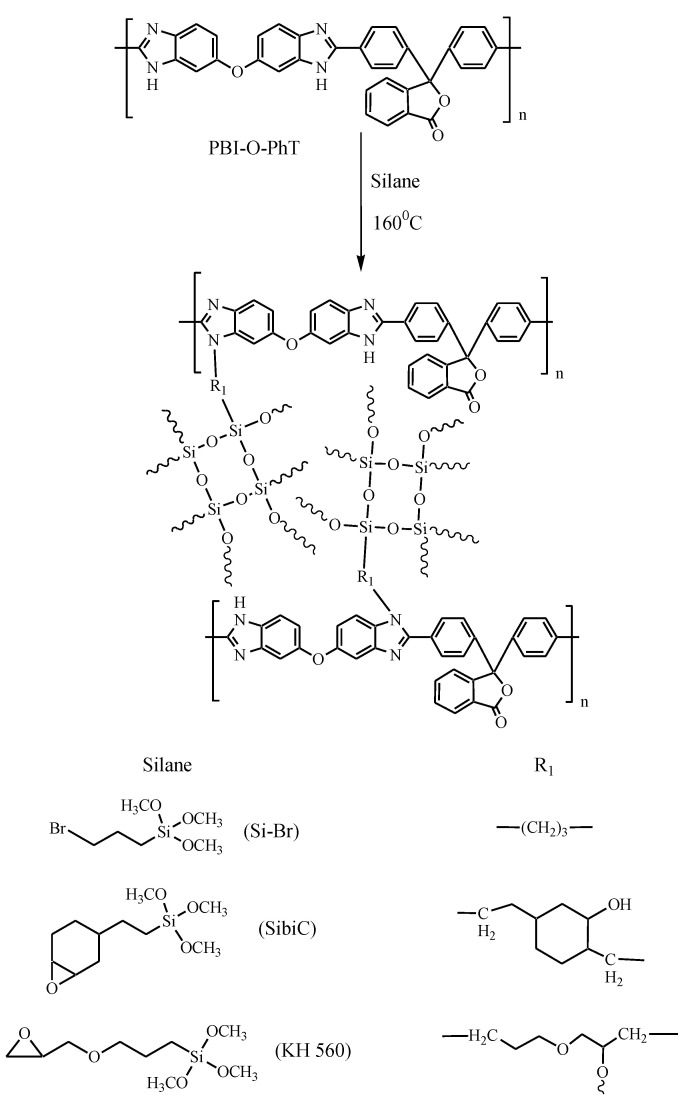
Scheme of the crosslinking reaction of polybenzimidazole with silanes of different structures.

The PBI-O-PhT polymer (Figure 1) for membrane preparation was synthesized from 4,4′-diphenylphthalidedicarboxylic acid and 3,3′,4,4′-tetraaminodiphenyl ether in Eaton’s reagent (P_2_O_5_:MeSO_3_H 9:1 *wt*/*wt*) according to [26]. The intrinsic viscosity [η] of the solution was found by extrapolating the reduced viscosity to a concentration, c = 0, in *N*-methylpyrrolidone (NMP) at 25 °C and was 2.02 dL/g, which corresponds to a molecular weight of ~90 kDa [26] (η_red_ = (t − t_0_)/ct_0_; where t and t_0_ are the solution and solvent flow times, respectively).

#### 2.1.2. Preparation of Crosslinked Membranes

Covalent crosslinking of polymer chains was performed using silanes of different structures, (3-Bromopropyl)trimethoxysilane (SiBr, Aldrich, St. Louis, MO, USA), trimethoxy [2-(7-oxabicyclo [4.1.0]hept-3-yl)ethyl]silane (SibiC, Aldrich) and (3-Glycidyloxypropyl)trimethoxysilane (KH 560, Aldrich) (Figure 1), using an in situ method. For this purpose, the calculated amount of silane was added to a solution of the polymer in *N*-methylpyrrolidone (N-MP, 4 g polymer/100 mL solvent) to obtain a crosslinking degree ranging from 1 to 50 mol%. The crosslinking degree was determined as the mole fraction of the PBI-O-PhT polymer chain units involved in the reaction with the crosslinking silane, assuming a 100% yield from the reaction. The mixture was then stirred for 24 h and dried on a glass substrate at 50–60 °C for three days. In the case of silane KH 560, a Teflon substrate was used due to the fact that the sample could not be separated from the glass without damage. To remove solvent residues and carry out the crosslinking reaction, the obtained films were heated in vacuum at 120 °C for 4 h and 160 °C for 1 h. Next, hydrolysis of the alkoxy groups of silanes was performed in 1M HCl solution at 80 °C for 24 h. After that, the films were washed from Cl^−^ ions in deionized water and dried in a vacuum at 120 °C. The thickness of the obtained films was 45 ± 5 mm. Thus, the films with silanol covalent crosslinking were obtained. Hereafter, these samples are referred to as PBI/silane-x, where x is the degree of crosslinking in mole fractions. The PBI-O-PhT membrane without silane addition was used as a comparison sample.

To ensure conductive properties, all the obtained samples were kept for seven days at 25 °C in phosphoric acid (conc. 75%). After that, the weight of the membranes increased 2.5–3 times. The obtained materials were dried in vacuum at 80 °C for 4 h and stored in a desiccator over P_2_O_5_.

#### 2.1.3. PBI/KH 560-Reinforced Membrane

*m*-PBI (Poly [2,2′-(m-phenylene)-5,5′-bibenzimidazole) was synthesized from isophthalic acid and 3,3′,4,4′-tetraaminobiphenyl in polyphosphoric acid as described in [27]. The intrinsic viscosity [η] was measured in 98% H_2_SO_4_ at 25 °C and was 0.80 dL g^−1^, which corresponds to a molecular weight ~30 kDa according to [28]. *m*-PBI nanofiber mat was prepared on an NS Lab setup (Elmarco, Liberec, Czech Republic) by the Nanospider^TM^ electrospinning technology (needle-free method from free liquid surface) from *m*-PBI solutions (15 wt.%) in the dimethylacetamide/ethanol mixture (9:1 *v*/*v*). Electrospinning was performed at 22 °C at a relative humidity of 25%. The electrospinning parameters were as follows: a voltage of +60/−35 kV (spinning electrode/collecting electrode), a current of 0.33–0.34 mA, a carriage speed of 4.0 s, an in/out air flow of 60/80 m^3^/h, an orifice size of 0.7 mm and a spinning distance of 180 mm. As a result, *m*-PBI nanofiber mats were obtained. The reinforced PBI/KH 560-0.3 membrane was obtained according to the analogous procedure for the PBI/KH 560-0.3 membrane by casting solution on the electrospun *m*-PBI mat.

### 2.2. Material Characterization

Microphotographs of the membrane cross-sections were obtained using a Hitachi SU-8000 (Hitachi, Tokyo, Japan) scanning electron microscope (SEM) with an accelerating voltage of 20 keV. The distribution of the Si element over the sample volume was investigated by energy dispersive X-ray spectroscopy. In order to obtain a cross-section, the films were pre-dipped in liquid nitrogen and then broken. Prior to the study, the samples were sprayed with chromium to prevent a charging effect during SEM imaging.

The thermal stability of the samples was studied by thermogravimetry (TGA) with differential scanning calorimetry (DSC) using a Netzsch STA 449 F1 with a heating rate of 5 K/min. Imaging was performed in helium with a flow rate of 20 mL/min.

The infrared absorption spectra of the samples were recorded on a Nicolet iS5 FTIR spectrometer (Thermo Fisher Scientific, Waltham, MA, USA) with an ATR accessory (diamond crystal) in the 4000–400 cm^−1^ region; the samples were prepared as thin films.

Since crosslinking significantly affects the physicochemical properties of the membranes, in particular their solubility, the crosslinking reaction was confirmed by a solubility test in *N*-methylpyrrolidone. The membrane samples were kept in N-MP at 80 °C for 24 h, after which the residue was dried at 100 °C for 24 h and weighed. The degree of dissolution of the samples was calculated from the mass loss during the experiment.

The oxidative stability of the membranes was evaluated by weight loss during treatment with the Fenton reagent (3% H_2_O_2_ solution with an Fe^2+^ content of 4 ppm). The dry membrane was kept in 50 mL of Fenton solution at 70 °C. Every 24 h, the membrane sample was taken out, washed and dried before weighing at 100 °C for 24 h. The membrane was then weighed and re-immersed in freshly prepared Fenton’s reagent solution to continue the testing.

After testing in Fenton’s reagent, a number of the samples were washed with HCl solution (~0.1 M), deionized water, dried and then immersed in phosphoric acid solution for doping. Before proton conductivity measurements, the samples were dried at 80 °C under vacuum conditions.

The phosphoric acid doping level (number of H_3_PO_4_ molecules per repeating polymer unit, n) of the membranes was determined based on the weight of the absorbed acid according to the method given in [29]. For this purpose, samples were weighed before and after acid doping and pre-dried from sorbed water. The water uptake for acid-doped membranes was determined by weighing the samples before and after drying.

Stress–strain curves were obtained using a Tinius Olsen H5KT (Tinius Olsen, Ltd., Redhill, UK) universal tensile device at 25 °C (sample size 50 × (9–10) mm^2^). The thickness and width of each sample were determined as the average of these values measured at five points. Five measurements were carried out for each sample. The strain rate was 2 mm/min.

The stabilization of phosphoric acid in the membranes was evaluated by holding the membrane over water vapor for 5 h. The samples were then dried at 100 °C for 24 h. The samples were weighed before and after the experiment, and the amount of acid that leaked from the membranes was estimated from this data.

Measurements of conductivity were carried out using an impedance meter, Z500 PRO (Elins, Chernogolovka, Russia), in the frequency range of 10–2 × 10^6^ Hz in potentiostatic mode with the amplitude of the sinusoidal signal being 80 mV with graphite electrodes. The ionic conductivity value was calculated by the extrapolation of semicircles of the volume component of conductivity to the axis of active resistances. Conductivity measurements at different relative humidities (RHs) and a constant temperature of 90 °C were performed in a Binder MKF115 (Binder, Neckarsulm, Germany) climatic chamber. The membranes were kept for 1 h at each RH value before measurement. The proton conductivity of air-dry membranes was studied in the temperature range 20–160 °C with a step of 10–15°. For all membranes, the fraction of electronic conductivity measured at a constant current did not exceed 0.01% of the total conductivity value.

The hydrogen permeability through the acid-doped membranes was studied by gas-liquid chromatography using a Crystallux-4000M (LLC Scientific and Production Company “Meta-chrome”, St. Petersburg, Russia) chromatograph with a thermal conductivity detector (30 mA current) and a packed column (5 Å Mole Seive sorbent, 2 m, 30 °C, 20 cm^3^ min^−1^, Ar) at two temperatures, 50 and 70 °C, with the gas streams pre-dehydrated by passing the gas through a U-shaped tube with 4 Å molecular sieves according to the procedure described in [30]. To produce hydrogen, we used a hydrogen generator produced by LLC Research and Production Association Khimelektronika, Russia. The experiment was performed in a thermostatted cell, in one part of which pure hydrogen was supplied and in the other part—argon at the rate of 20 mL min^−1^. The hydrogen permeability coefficient *P* (cm^2^ s^−1^) was calculated by the formula:

(1) $$P=\frac{jL}{C_{H}-C_{Ar}},$$

where *L* is the membrane thickness, *C_H_* is the average volume concentration of hydrogen in the chamber where hydrogen was supplied and *C_Ar_* is the average volume concentration of hydrogen in the chamber where argon was supplied. The gas flow through the membrane *j* was calculated from the ratio:

(2) $$j=\frac{C_{Ar}V_{t}}{S},$$

where *C_Ar_* is the average volume concentration of hydrogen in the chamber in which argon was supplied, reduced to normal conditions, *V_t_* is the volume flow rate of the carrier gas and *S* is the active area of the membrane.

The membrane electrode assemblies (MEAs) were placed in standard Arbin Instruments testing cells (College Station, TX, USA) with two graphite flow field plates, with a working electrode area of 5 cm^2^. High-temperature polymer electrolyte membrane (HT-PEM) fuel cell MEAs were operated with typical Celtec^®^-P 1000 MEA (BASF, Florham Park, NJ, USA) anode and cathode [31]. Fuel cell operations were carried out at 150 °C and 200 °C. Hydrogen was supplied to the anode at a rate of 100 mL/min using a GVCh-6 Khimelektronika hydrogen generator (LLC Research and Production Association Khimelektronika, Moscow, Russia). Air was supplied at a rate of 600 mL/min to the cathode. Hydrogen and air were supplied without additional humidification. Polarization curves in the range 0.95–0.02 V were obtained using a P-150X potentiostat-galvanostat (Electrochemical Instruments, Chernogolovka, Russia).

## 3. Results and Discussion

One of the methods for determining the crosslinking reaction and the subsequent stabilization of the membranes is measuring the change in mass during prolonged exposure in a solvent with a high affinity for the polymer (solubility test) [23]. N-MP was used as such a solvent. The experimental results are shown in Table 1. The non-crosslinked PBI polymer dissolves completely. In the case of SiBr and KH 560 silanes, even at 1% in terms of crosslinking degree, the film does not dissolve completely. For SibiC membrane, the stabilization begins at a 10% crosslinking degree. It is most likely that the latter case can be attributed to an incomplete reaction yield in the case of the bulk substituent (oxabicyclo [4.1.0]heptyl) due to steric difficulties in the reaction with the polymer chain containing the bulk phthalate group. For silanes with linear substituents, the reaction is more productive.

**Figure 2:**
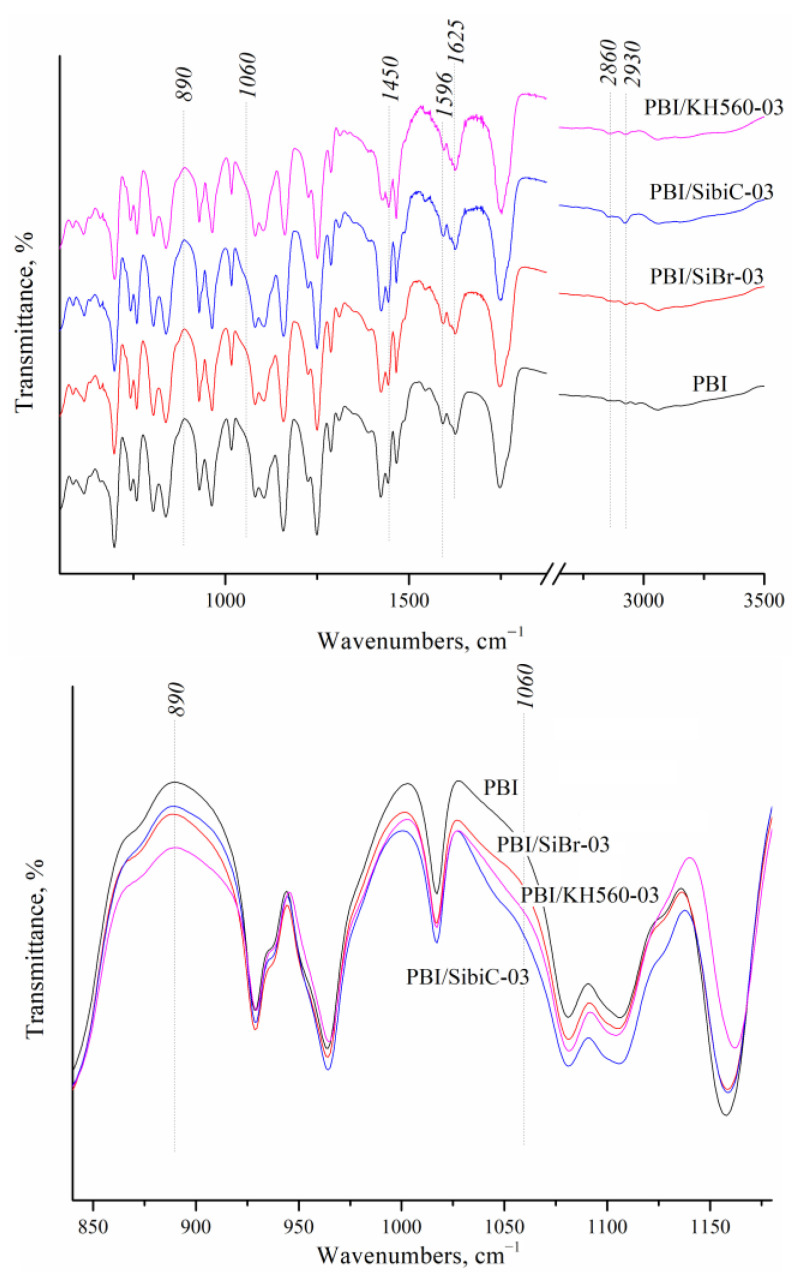
FTIR-spectra of crosslinked PBI membranes with different silanes.

According to **IR spectroscopy** (Figure 2) after crosslinking, the bands which refer to C-Br strain vibrations (in the region of 660 cm^−1^) in the case of PBI/SiBr or to the epoxy group (910 cm^−1^) for SibiC and KH 560 are absent in the membrane spectra. In the region of 2930 and 2850 cm^−1^ for crosslinked membranes, bands belonging to symmetric and asymmetric –CH_2_– vibrations appear. The bands of the valent vibrations of the C=N-groups of the imidazole ring are in the region of 1625 cm^−1^, and those of the benzene ring are near 1450 and 1596 cm^−1^. In all systems, in the region of 1060–1100 cm^−1^ and 890 cm^−1^, an increase in the intensity of the band belonging to the phthalate group of the polymer chain is observed due to overlapping with the bands belonging to the stretching vibrations of the Si–O–Si and Si–OH bonds. These changes confirm the successful crosslinking and formation of the siloxane bond network shown in Figure 1.

**Figure 3:**
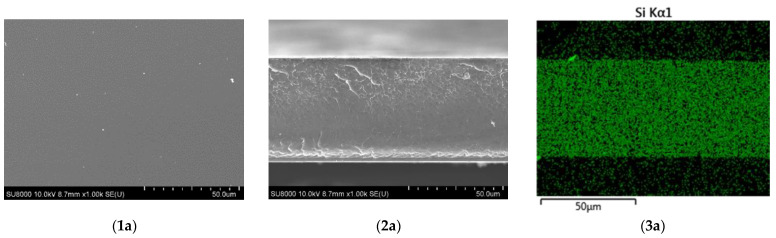

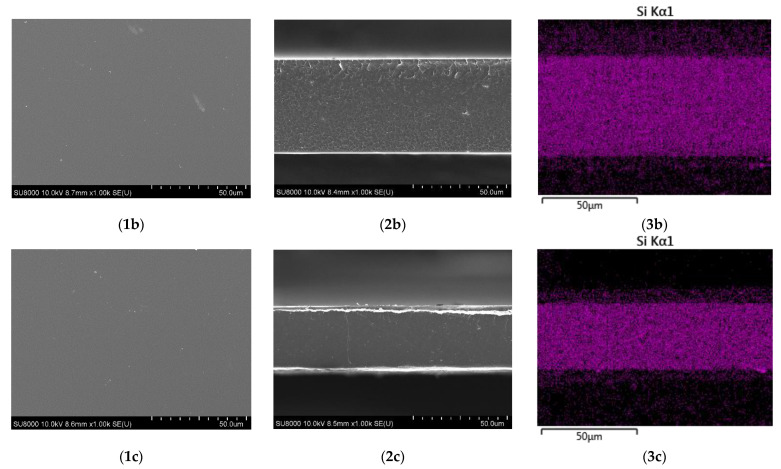
SEM images of the surface (**1**); cross-section (**2**) and Si mapping (**3**) of PBI/SiBr-0.5 (**a**); PBI/SibiC-0.5 (**b**) and PBI/KH 560-0.5 (**c**).

According to the **SEM** EDX data (Figure 3), all the obtained samples have a dense structure without porosity, which is important for the use of membranes in fuel cells. X-ray microanalysis data confirm the homogeneity of the samples and uniform distribution of silicon throughout the film volume. It should be noted that the authors of [25] observed the formation of micropores in the structure of membranes after silanol crosslinking, which they attributed to the escape of alcohol and water molecules after the hydrolysis of the alkoxy groups of silanes. In our case, the membranes remained dense and homogeneous, which was probably due to the drying conditions after hydrolysis.

**Figure 4:**
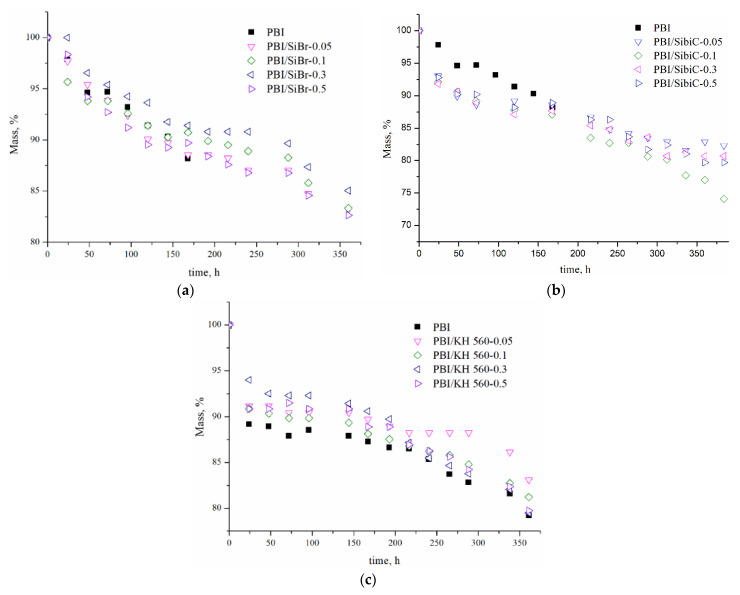
Results of degradation of cross-linked PBI membranes in the Fenton test. SiBr (**a**), SibiC (**b**) and KH 560 (**c**) were used as crosslinking reagents.

**Oxidative stability** is an important characteristic for polymer electrolytes and affects the durability of the material. It is usually evaluated by weight loss after testing the samples in Fenton’s reagent (3% H_2_O_2_ solution in the presence of 4 ppm Fe^2+^). As can be seen in Figure 4, after crosslinking with SiBr and CH 560, the membranes show increased stability compared to the reference sample. For example, after 168 h of testing, the comparison sample loses 12 wt.% and both PBI/SiBr at x = 0.1–0.3 and PBI/KH 560 at x = 0.3–0.5 roughly 9 wt.% (Figure 4). A smaller mass loss is observed for samples with 30% crosslinking. At the same time, when SibiC is used as a crosslinking reagent, the mass loss in the first few days is even slightly greater than for the non-crosslinked sample (in the first 48 h ~10 wt.% for all crosslinking degrees compared to 5.5% for the reference sample). However, all crosslinked samples retain their integrity even after 360 h of processing, while the non-crosslinked sample cracks completely after 192 h.

Most probably, with regards to the material, crosslinking provides less permeability to oxidizing substances. At the same time, in the case of SiBr and KH 560, a slightly higher stability is observed compared to the silane with a bulk group. This is due to a lower crosslinking degree in the case of SibiC due to the incomplete reaction. In addition, with a larger functional group, the forming structure may be less dense, which makes the polymer chains more accessible to the oxidant.

The swelling and dimensional stability of samples were determined as the ratio of thickness and area, respectively, before and after acid doping. Crosslinking in all cases leads to a slight decrease in membrane swelling (33% for the initial membrane and 30 ± 1% for crosslinked samples). The best dimensional stability is observed for the SiBr crosslinked samples: the increase in sample area after acid doping decreases from 38% to 32% for x = 0.5 and to 36% and 34% for silanes SibiC and KH 560, respectively.

**Figure 5:**
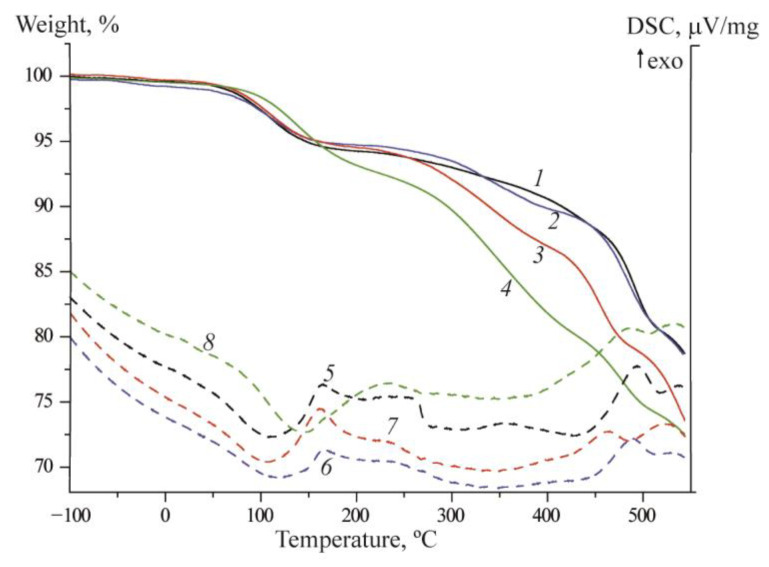
Thermal stability of initial PBI (1, 5), PBI/SiBr (2, 6), PBI/SibiC (3, 7) and PBI/KH 560 (4, 8) membranes with a crosslinking degree of 0.3.

On the thermograms of the studied samples, obtained by using the **DSC** method, the first stage of mass loss associated with the loss of sorbed water is observed at 60–140 °C (Figure 5). In the case of membranes with silane KH 560, dehydration occurs at slightly higher temperatures. The second step, associated with the thermal decomposition of PBI, this begins around 270 °C. For crosslinked polymers with SibiC and KH 560, this temperature is almost the same, while for SiBr it rises to 310 °C. However, the mass loss for crosslinked samples turns out to be greater due to the simultaneous decomposition of the organic silane substituent and the destruction of polysiloxane bonds. Thus, in the case of crosslinking with the SiBr silane, an improvement in thermostability is observed due to the formation of a more strongly “crosslinked” structure.

**The phosphoric acid doping level** (n, number of H_3_PO_4_ molecules per repeating unit of polymer) of the membranes is shown in Table 2. In the case of the silane with a bulk substituent (SibiC), the acid doping level decreases with an increasing degree of crosslinking, in contrast to the silanes with flexible linear substituents. This may be due to a decrease in the free volume inside the polymer. In addition, an increase in the siloxane concentration leads to the sorption of an additional amount of acid due to electrostatic interactions. Similar data were obtained in [17,25] for PBI crosslinked with silanes with linear substituents. On the other hand, the formation of a network of siloxane bonds by silanes with flexible non-bulky substituents can increase the distance between polymer chains, which can be filled with additional acid.

The water uptake of the membranes changes in a similar fashion (it increases by 1.5–2% for samples crosslinked with SiBr and KH 560 silanes and decreases by 1.3–2% for PBI/SibiC samples), which is primarily due to changes in the content of hygroscopic phosphoric acid in the membrane.

Concerning the operation of PBI membranes in the FC, an important problem is the loss of conductivity due to acid leaching by water contained in the feed gases or formed during FC operation. In order to determine the effect of crosslinking on the membrane’s ability to retain acid, an **acid leaching test** was performed. For this purpose, the membrane samples were held over water vapor for 5 h. After that, they were dried and weighed. In this way, the amount of phosphoric acid removed from the membrane with moisture was determined (Table 2). The initial membrane loses practically all acid after such treatment, which is generally in agreement with the data in the literature [23]. Samples with the silanol crosslinking show much better acid retention. The PBI/KH 560–0.5 sample showed the best result. The results prove that the crosslinking of membranes is an effective method for stabilizing phosphoric acid in their matrix and reducing its leaching into the membrane environment. The authors [23] attribute this phenomenon to the amount of water that is sorbed by the membrane matrix. However, it is more plausible that the mobility of phosphoric acid is reduced due to crosslinking, which limits its diffusion.

**The mechanical properties** of membranes are an important characteristic for creating long-life FCs. Studies of the mechanical properties of membranes undoped with phosphoric acid have shown that silane crosslinking with SiBr and SibiC silanes does not lead to a significant change in Young’s modulus, while for silane KH 560 there is a slight decrease (Table 3). The proportional limit stress increases for samples crosslinked with the SiBr and SibiC silanes and does not change for the PBI/KH 560 compared with the reference sample. In addition, the crosslinking leads to an increase in tensile strength. The break strain for the undoped sample and PBI/SiBr is 7 ± 1 and 10 ± 1%, respectively, and for membranes with SibiC and KH 560 silanes it is 15 ± 1 and 13 ± 2%, respectively. The reason for this may be the disorder of the packaging with crosslinking agents.

**Figure 6:**
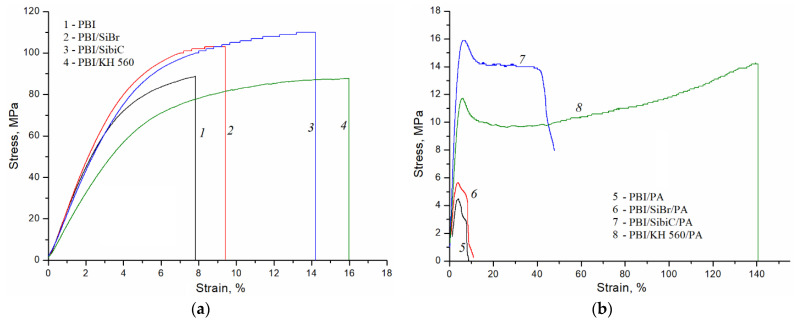
Stress–strain curves of undoped (**a**) and acid-doped (**b**) PBI (1, 5), PBI/SiBr-0.1 (2, 6), PBI/SibiC-0.1 (3, 7) and PBI/KH 560 (4, 8).

Doping with phosphoric acid leads to a significant decrease in membrane strength and elasticity by increasing the distance between the main polymer chains. This leads to a decrease in intermolecular interactions, in particular, hydrogen bonds between –N= and –NH– groups. In fact, phosphoric acid in these materials plays the role of a plasticizer. This phenomenon becomes especially significant at a high degree of phosphoric acid doping. From the strain curves for such membranes, it can be seen that after the first reversible stretching, a relaxation occurs in the polymer, leading to a decrease in stress (Figure 6). At the same time, a “neck” begins to form in the sample, which in the case of silanes SibiC and KH 560 is further stretched without a significant increase in stress. This is the stage of highly elastic deformation, which is brought about due to an increase in mobility and the possibility of conformational changes in polymer chains. At the same time, especially for acid-doped samples, the silane crosslinking contributes to a significant improvement in the mechanical characteristics of the membranes compared to the initial sample. Membranes with the SibiC silane, which have a slightly lower degree of acid doping, possess higher Young’s modulus and proportional limit stress values. The crosslinking effect can be evaluated by comparing membranes with close phosphoric acid content [17]. Membranes with SiBr and KH 560 with equal crosslinking degree show similar acid doping level values in comparison with each other and higher values compared with the initial sample. For them, an increase in the strength characteristics with a growing crosslinking degree is observed despite a higher acid content. This is even more so the case for the membranes crosslinked with silane KH 560. The results indicate that the network of siloxane bonds is able to harden the acid-doped membranes even with an increase in the doping level. It is supposed that this phenomenon is related to the fact that, in general, the reduction of intermolecular interactions between the polymer chains occurs due to the presence of free phosphoric acid in the membrane, which destroys the hydrogen bonds between the chains. At the same time, phosphoric acid is not able to destroy siloxane bonds, and in the presence of acid they determine the mechanical properties of the membranes. The values of tensile strength and elongation are not given for the acid-doped membranes, because, as can be seen from the curves, the membranes withstand a much higher stress before reaching the highly elastic state before rupture. In this case, in our opinion, it is more correct to compare the proportional limit stress values, since it is after elongation and up to that point that relaxation phenomena begin to occur.

**Figure 7:**
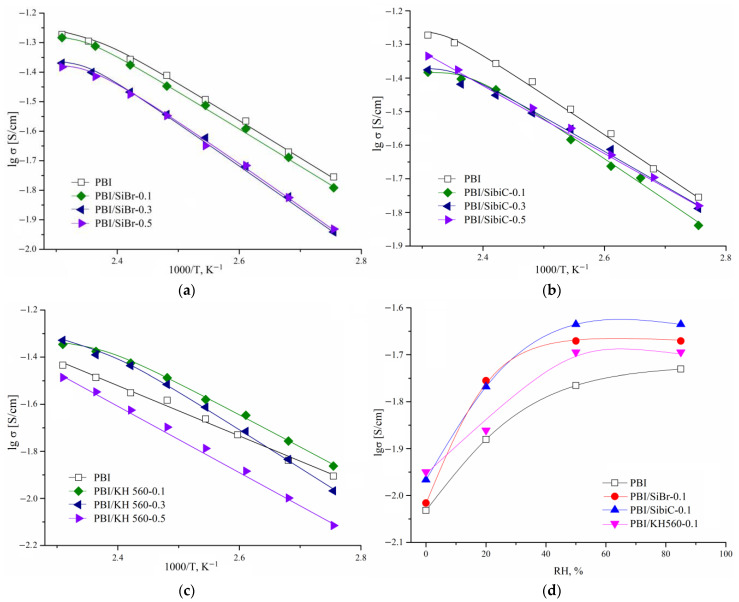
Dependence of conductivity on temperature (**a**–**c**) and relative humidity; (**d**) for PBI membranes crosslinked with silanes of different structure.

One of the most important properties affecting the capacity of fuel cells based on polyelectrolyte membranes is the **proton conductivity**. Its values may depend on operating conditions such as temperature and relative humidity. High conductivity values indicate that the amount of embedded acid is sufficient to maintain high conductivity due to the formation of a network of hydrogen bonds through which proton transfer occurs by the Grotthuss mechanism. It can be noted that the conductivity of all samples changes insignificantly relative to the reference sample by no more than 0.1 order of magnitude (Figure 7). In the case of membranes crosslinked with silane KH 560, the dependence of conductivity on the crosslinking degree passes through a maximum. At 160 °C, the PBI/KH 560-0.3 sample shows the maximum value of conductivity (47 mS/cm). For the reference sample, the conductivity is 37 mS/cm at this temperature. At a high crosslinking degree, the conductivity decreases, which is associated with both the fact that the structure becomes more rigid and the fact that the nitrogen atoms of the imidazole ring associated with the organic substituent of silane do not participate in proton transfer processes. It is likely that, in the case of silane KH 560, a slightly higher mobility of polymer chains is preserved due to the longer chain of the substituent, resulting in an increase in conductivity. In addition, as noted above, the diffusion mobility of phosphoric acid is reduced in crosslinked membranes. However, this does not lead to a sharp drop in conductivity because the translational mobility of large phosphate anions is not crucial for the realization of proton conductivity, which requires only their rotational mobility and proton hopping [32,33]. In the case of linear silanes SiBr and KH560, the conductivity activation of the non-crosslinked sample, 21.2 ± 0.9 kJ/mol, increases with an increase in crosslinking degree, and for samples with the crosslinking degree of 0.5 the activation energy is 24.2 ± 0.9 and 27.0 ± 0.6 kJ/mol, respectively. In the case of crosslinking using SibiC silane with a bulk substituent, by contrast, the activation energy decreases and is 19.1 ± 0.5 kJ/mol for a cross-linking degree of 0.5. This may suggest the formation of a less dense structure in the presence of bulk silane fragments, in which the proton transfer takes place with a lower activation barrier. It should be noted that at high temperatures there is a deviation from the Arrhenius dependence regarding conductivity, which may be due to the gradual dehydration of the samples.

There is also an increase in conductivity with increasing relative humidity, which is associated with the hydration of the membranes. Thus, the concentration of electric carriers grows due to the increase in the degree of acid dissociation [34]. Additionally, water can be incorporated into the system of hydrogen bonds formed by phosphoric acid molecules and basic nitrogen atoms of the polymer chain along which proton transfer occurs, thus increasing the number of proton transfer centers. In addition, the viscosity of the solution inside the polymer decreases, which also facilitates proton transfer. The sharpest increase in conductivity is observed at low RH values. An increase in humidity above 50% does not lead to a noticeable increase in conductivity because in this case the membrane loses a noticeable portion of the bound phosphoric acid.

**Figure 8:**
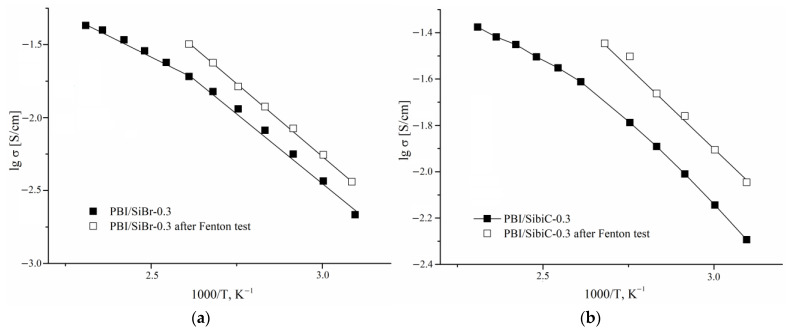

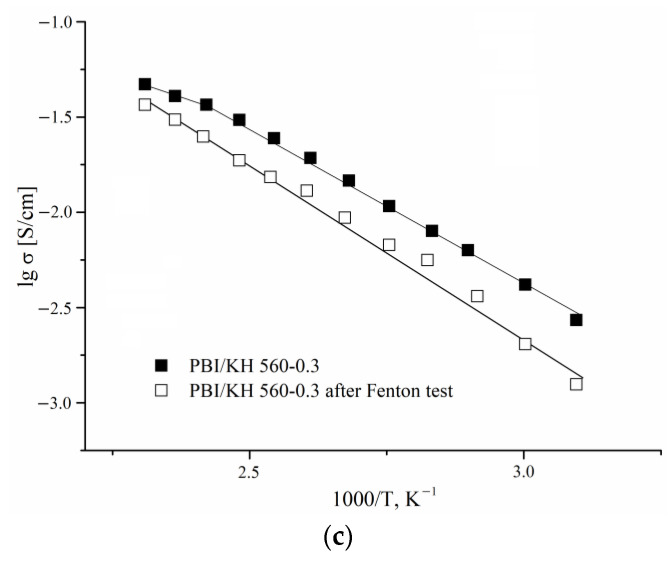
Conductivity temperature dependence for membrane samples after testing under oxidizing conditions with Fenton’s reagent. SiBr (**a**), SibiC (**b**) and KH 560 (**c**) were used as crosslinking reagents. For comparison, the data of samples not treated with Fenton’s reagent are given.

To determine **the degradation of the transport properties** of the membranes, their conductivity was also measured after 360 h of treatment with Fenton’s reagent. After oxidative stability testing, the samples were also treated with hydrochloric acid solution to remove iron oxide and washed with deionized water. After that, the samples were doped with phosphoric acid for further conductivity measurements. Figure 8 shows the temperature dependences in terms of conductivity for samples with a sufficiently high degree of crosslinking which retained their integrity and did not crack. A crosslinking degree of 0.3 was chosen as an example; other samples had similar changes in properties. Membranes with the SiBr and SibiC silanes that passed oxidative testing begin to degrade above 110–120 °C. At the same time, the crosslinked KH 560 membranes keep well, despite lengthy oxidant treatment (360 h). It can be noted that the conductivity of the crosslinked membranes after such treatment changes insignificantly. The observed increase in the conductivity of the SiBr and SibiC crosslinked membranes can be explained by the appearance of additional proton transfer pathways. According to the study of the mechanism of oxidative degradation in [35], treatment with Fenton’s reagent results in the oxidation of the polymer chain sections with the formation of C–OH bonds as well as the opening of imidazole cycles due to the hydrolysis of C–N/C=N bonds, accompanied by the fragmentation of the polymer chain into shorter molecules. At the same time, in the case of KH 560, crosslinking probably “works”, so that the main chain becomes less accessible to the oxidant and better preserved, most likely by the formation of a denser membrane structure. This is confirmed both by the preservation of the mechanical integrity of the membrane and by a less significant change in conductivity. In terms of oxidative degradation, the best results were obtained using silane KH 560.

**Gas permeability** also plays an important role in the application of membranes in FCs. It determines the fuel transfer through the membrane that is not accompanied by energy production. A study of the hydrogen permeability of acid-doped membranes showed that, in the case of silanes SiBr and KH 560, there is a decrease in hydrogen diffusion permeability, which becomes significant only at high crosslinking degrees (Table 4). At the same time, for SibiC, an increase in hydrogen permeability is observed. This can be explained by the denser structure formed when using the silanes with linear substituents, which agrees with the other data given above.

**Figure 9:**
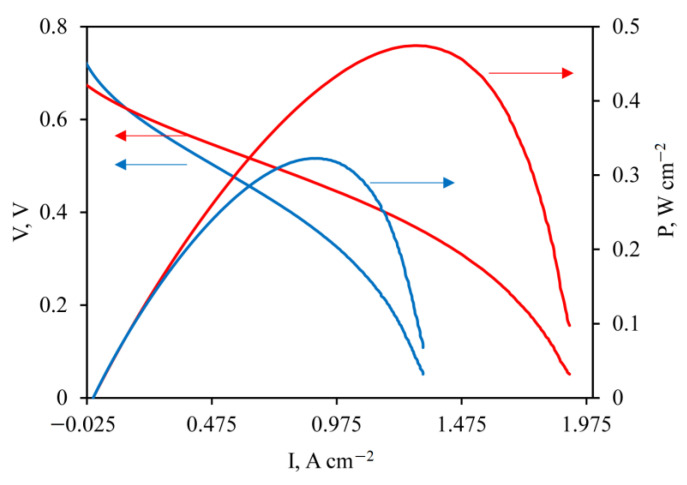
Polarization curves and power density data for MEA with the reinforced PBI/KH 560-0.3 membrane at 150 °C (blue) and 200 °C (red).

The polarization curves and power density data for **membrane electrode assemblies** (MEAs) with the PBI/KH 560 membrane at 150 and 200 °C are shown in Figure 9. The performance of the HT-PEM fuel cell MEA with the PBI/KH 560 membrane reaches 0.474 W/cm^2^ at 1.3 A/cm^2^ at 200 °C. This data demonstrates that the crosslinked membranes can be used in fuel cells.

## 4. Conclusions

The silanol crosslinked PBI membranes obtained possess greater morphological stability under radical oxidation conditions due to the formation of a network of siloxane bonds and a denser structure, which hinders the penetration of oxidizing agents. In some cases, the degree of doping with phosphoric acid increases, and crosslinking helps to improve the mechanical strength of doped membranes, which is important when they are used as polyelectrolyte membranes in fuel cells. The optimal degree of crosslinking in terms of the influence on the whole complex of properties is x = 0.1–0.3. In this case, a sufficient number of basic nitrogen atoms participating in proton transfer is preserved and there is no appreciable loss of conducting properties. The reinforced membranes [36] prepared with silanol and m-PBI were found to be suitable for HT-PEM fuel cells.

## Figures and Tables

**Table 1:** Residual mass (%) after solubility test in *N*-methylpyrrolidone.

Crosslinking Degree	SiBr	SibiC	KH 560
0	0		
0.01	17.3	0	58.6
0.03	53.9	0	82.7
0.05	83.2	0	95.8
0.1	90.9	55.1	97.0
0.3	100.0	92.3	98.6
0.5	100.0	96.5	98.0

**Table 2:** Phosphoric acid doping level of membranes (*n*) and acid leaching (%) in relation to the silane used for crosslinking and the degree of crosslinking.

Crosslinking Degree	Acid Doping Level (*n*)			Acid Leaching (%)		
	SiBr	SibiC	KH 560	SiBr	SibiC	KH 560
0 (initial PBI)	9.7			96		
0.01	9.7	9.7	9.7	-	-	-
0.03	9.7	9.7	9.8	-	-	-
0.05	9.7	9.7	9.9	70	76	86
0.1	10.1	9.6	10.2	79	71	86
0.3	10.1	9.3	10.2	-	-	-
0.5	10.2	8.9	9.9	84	79	63

**Table 3:** The mechanical properties of PBI and crosslinked membranes before and after doping with phosphoric acid.

Sample	Without Acid			After Acid Doping	
	Young’s Modulus, MPa	Proportional Limit Stress, MPa	Tensile Strengh, MPa	Young’s Modulus, MPa	Proportional Limit Stress, MPa
PBI	24.9 ± 0.8	80 ± 4	91 ± 3	1.7 ± 0.4	5.5 ± 0.9
PBI/SiBr-0.1	26 ± 1	98 ± 5	104 ± 1	1.8 ± 0.5	5.7 ± 1
PBI/SiBr-0.5	23 ± 1	93 ± 5	104 ± 4	3.8 ± 0.4	11.4 ± 0.1
PBI/SibiC-0.1	22.4 ± 0.4	100 ± 3	109 ± 1	4.1 ± 0.2	16 ± 1
PBI/SibiC-0.5	22.6 ± 0.8	105 ± 2	116 ± 2	4.5 ± 0.6	20 ± 3
PBI/KH 560-0.1	17.1 ± 0.5	79 ± 2	90 ± 4	3.3 ± 0.2	11.6 ± 0.4
PBI/KH 560-0.5	18 ± 1	79 ± 2	96 ± 2	5 ± 1	11.9 ± 0.8

**Table 4:** Diffusion permeability (cm^2^ s^−1^) of hydrogen through PBI membranes crosslinked with different silanes.

Crosslinking Degree	RH, %	SiBr	SibiC	KH 560
0	50	(2.8 ± 0.6) × 10^−9^		
	70	(7.0 ± 0.4) × 10^−9^		
0.1	50	(2.2 ± 0.4) × 10^−9^	(4.4 ± 0.7) × 10^−9^	(2.3 ± 0.4) × 10^−9^
	70	(5.7 ± 0.7) × 10^−9^	(1.14 ± 0.03) × 10^−8^	(4.6 ± 0.8) × 10^−9^
0.5	50	(2.8 ± 0.2) × 10^−9^	(4.0 ± 0.4) × 10^−9^	(3.9 ± 0.4) × 10^−9^
	70	(5.8 ± 0.2) × 10^−9^	(7.8 ± 1.2) × 10^−9^	(5.9 ± 0.5) × 10^−9^

## Data Availability

Not applicable.

## References

[1] Yaroslavtsev A.B., Stenina I.A., Golubenko D.V. (2020). Membrane materials for energy production and storage. Pure Appl. Chem..

[2] Sun C., Negro E., Nale A., Pagot G., Vezzù K., Zawodzinski T.A., Meda L., Gambaro C., Di Noto V. (2021). An efficient barrier toward vanadium crossover in redox flow batteries: The bilayer [Nafion/(WO_3_)_x_] hybrid inorganic-organic membrane. Electrochim. Acta.

[3] Agmon N. (1995). The Grotthuss Mechanism. Chem. Phys. Lett..

[4] Moore R.B., Mauritz K.A. (2004). State of Understanding of Nafion. Chem. Rev..

[5] Kalathil A., Raghavan A., Kandasubramanian B. (2019). Polymer Fuel Cell Based on Polybenzimidazole Membrane: A Review. Polym. Plast. Technol. Mater..

[6] Filippov S.P., Yaroslavtsev A.B. (2021). Hydrogen energy: Development prospects and materials. Russ. Chem. Rev..

[7] Li Q., Jensen J.O., Savinell R.F., Bjerrum N.J. (2009). High temperature proton exchange membranes based on polybenzimidazoles for fuel cells. Prog. Polym. Sci..

[8] Yaroslavtsev A.B., Stenina I.A., Kulova T.L., Skundin A.M., Desyatov A.V., Andrews D.L., Nann T., Lipson R.H. (2019). Nanomaterials for Electrical Energy Storage. Comprehensive Nanoscience and Nanotechnology, V.5 Applications of Nanoscience.

[9] Escorihuela J., Olvera-Mancilla J., Alexandrova L., Castillo L.F., Compañ V. (2020). Recent Progress in the Development of Composite Membranes Based on Polybenzimidazole for High Temperature Proton Exchange Membrane (PEM) Fuel Cell Applications. Polymers.

[10] Haider R., Wen Y., Ma Z.-F., Wilkinson D.P., Zhang L., Yuan X., Song S., Zhang J. (2021). High temperature proton exchange membrane fuel cells: Progress in advanced materials and key technologies. Chem. Society Rev..

[11] Wang X., Wang D., Wang S., Li J., Liu G., Cui Y., Liang D., Wang X., Yong Z., Wang Z. (2022). High-Performance Proton Exchange Membranes Based on Block Polybenzimidazole and Organic-Inorganic Fillers with a Low Acid Doping Level. ACS Appl. Energy Mater..

[12] Xiao Y., Ma Q., Shen X., Wang S., Xiang J., Zhang L., Cheng P., Du X., Yin Z., Tang N. (2022). Facile preparation of polybenzimidazole membrane crosslinked with three-dimensional polyaniline for high-temperature proton exchange membrane. J. Power Sources.

[13] Qu E., Hao X., Xiao M., Han D., Huang S., Huang Z., Wang S., Meng Y. (2022). Proton exchange membranes for high temperature proton exchange membrane fuel cells: Challenges and perspectives. J. Power Sources.

[14] Peng J., Fu X., Liu D., Luo J., Wang L., Peng X. (2022). An effective strategy to enhance dimensional-mechanical stability of phosphoric acid doped polybenzimidazole membranes by introducing in situ grown covalent organic frameworks. J. Membr. Sci..

[15] Lysova A.A., Ponomarev I.I., Yaroslavtsev A.B. (2019). Effect of the nature of functional groups grafted on the surface of silica nanoparticles on properties of the hybrid proton-conductive membranes based on N-phosphorylated polybenzimidazole. Mendeleev Commun..

[16] Guo Z., Perez-Page M., Chen J., Ji Z., Holmes S.M. (2021). Recent advances in phosphoric acid–based membranes for high–temperature proton exchange membrane fuel cells. J. Energy Chem..

[17] Yang J., Gao L., Wang J., Xu Y., Liu C., He R. (2017). Strengthening Phosphoric Acid Doped Polybenzimidazole Membranes with Siloxane Networks for Using as High Temperature Proton Exchange Membranes. Macromol. Chem. Phys..

[18] Yang J., Xu Y., Liu P., Gao L., Che Q., He R. (2015). Epoxides cross-linked hexafluoropropylidene polybenzimidazole membranes for application as high temperature proton exchange membranes. Electrochim. Acta.

[19] Yang J., Li Q., Cleemann L.N., Jensen J.O., Pan C., Bjerrum N.J., He R. (2013). Crosslinked hexafluoropropylidene polybenzimidazole membranes with chloromethyl polysulfone for fuel cell applications. Adv. Energy Mater..

[20] Wang L., Liu Z., Liu Y., Wang L. (2019). Crosslinked polybenzimidazole containing branching structure with no sacrifice of effective N-H sites: Towards high-performance high-temperature proton exchange membranes for fuel cells. J. Membr. Sci..

[21] Yang J., Jiang H., Gao L., Wang J., Xu Y., He R. (2018). Fabrication of crosslinked polybenzimidazole membranes by trifunctional crosslinkers for high temperature proton exchange membrane fuel cells. Int. J. Hydrogen Energy.

[22] Harilal, Nayak R., Ghosh P.C., Jana T. (2020). Cross-Linked Polybenzimidazole Membrane for PEM Fuel Cells. ACS Appl. Polym. Mater..

[23] Özdemir Y., Özkan N., Devrim Y. (2017). Fabrication and Characterization of Cross-linked Polybenzimidazole Based Membranes for High Temperature PEM Fuel Cells. Electrochim. Acta.

[24] Kerres J., Atanasov V. (2015). Cross-linked PBI-based high-temperature membranes: Stability, conductivity and fuel cell Performance. Int. J. Hydrogen Energy.

[25] Wang S., Zhao C., Ma W., Zhang N., Zhang Y., Zhang G., Liu Z., Na H. (2013). Silane-cross-linked polybenzimidazole with improved conductivity for high temperature proton exchange membrane fuel cells. J. Mater. Chem. A.

[26] Fomenkov A.I., Blagodatskikh I.V., Ponomarev I.I., Volkova Y.A., Ponomarev I.I., Khokhlov A.R. (2009). Synthesis and molecular mass characteristics of some cardo poly(benzimidazoles). Polym. Sci. Ser. B.

[27] Iwakura Y., Uno K., Imai Y. (1964). Polyphenylenebenzimidazoles. J. Polym. Sci. A.

[28] Yuan Y., Johnson F., Cabasso I. (2009). Polybenzimidazole (PBI) molecular weight and Mark-Houwink equation. J. Appl. Polym. Sci..

[29] Lysova A.A., Ponomarev I.V.I., Volkova Y.A., Ponomarev I.I., Yaroslavtsev A.B. (2018). Effect of Phosphorylation of Polybenzimidazole on Its Conductive Properties. Pet. Chem..

[30] Lysova A.A., Stenina I.A., Volkov A.O., Ponomarev I.I., Yaroslavtsev A.B. (2019). Proton conductivity of hybrid membranes based on polybenzimidazoles and surface-sulfonated silica. Solid State Ion..

[31] Schmidt T.J., Baurmeister J. (2008). Properties of high-temperature PEFC Celtec^®^-P 1000 MEAs in start/stop operation mode. J. Power Sources.

[32] Søndergaard T., Cleemann L.N., Becker H., Aili D., Steenberg T., Hjuler H.A., Seerup L., Li Q., Jensen J.O. (2017). Long-term durability of HT-PEM fuel cells based on thermally cross-linked polybenzimidazole. J. Power Sources.

[33] Stenina I.A., Yaroslavtsev A.B. (2021). Ionic Mobility in Ion-Exchange Membranes. Membranes.

[34] Lysova A.A., Yaroslavtsev A.B. (2019). New Proton-Conducting Membranes Based on Phosphorylated Polybenzimidazole and Silica. Inorg. Mater..

[35] Liao J.H., Li Q.F., Rudbeck H.C., Jensen J.O., Chromik A., Bjerrum N.J., Kerres J., Xing W. (2011). Oxidative Degradation of Polybenzimidazole Membranes as Electrolytes for High Temperature Proton Exchange Membrane Fuel Cells. Fuel Cells.

[36] Ponomarev I.I., Skupov K.M., Modestov A.D., Lysova A.A., Ponomarev I.I., Vtyurina E.S. (2022). Cardo polybenzimidazole (PBI-O-PhT) based membrane rein-forced with m-polybenzimidazole electrospun nanofiber mat for HT-PEM fuel cell applications. Membranes.

